# Geriatric assessment-based therapy for older patients with diffuse large B-cell lymphoma: results of a two-decade clinical experience

**DOI:** 10.1186/s12885-026-16039-6

**Published:** 2026-04-25

**Authors:** Hironao Nukariya, Shinsaku Washinosu, Haruna Nishimaki-Watanabe, Takashi Ichinohe, Shun Ito, Toshihide Endo, Kazuya Kurihara, Yuichi Takeuchi, Takashi Koike, Takashi Hamada, Shimon Otake, Hiromichi Takahashi, Masaru Nakagawa, Noriyoshi Iriyama, Daisuke Tsutsumi, Akihiro Uchiike, Tatsuya Hayama, Hideki Nakamura, Katsuhiro Miura

**Affiliations:** 1 Division of Hematology and Rheumatology, Department of Medicine, Nihon University School of Medicine, Tokyo, Japan; 2 Tumor Center, Nihon University Itabashi Hospital, 30-1 Oyaguchikamicho, Itabashi City, Tokyo, 173-8610 Japan; 3 Division of Oncologic Pathology, Department of Pathology and Microbiology, Nihon University School of Medicine, Tokyo, Japan; 4 Department of Hematology and Rheumatology, NHO Saitama Hospital, Saitama, Japan; 5 Department of Hematology, Kasukabe Medical Center, Saitama, Japan; 6 Department of Internal Medicine, Saitama Coopelative Hospital, Saitama, Japan

**Keywords:** Albumin, Comorbidity, Diffuse large B-cell lymphoma, Geriatric assessment, Older patients, R-CHOP

## Abstract

**Background:**

Treating older patients with diffuse large B-cell lymphoma (DLBCL) is challenging due to heterogeneity in fitness and tolerance to chemotherapy with rituximab, cyclophosphamide, doxorubicin, vincristine, and prednisolone (R-CHOP). Thus, we previously established the Age, Comorbidity, and Albumin (ACA) index for this population, a geriatric assessment comprising four categories. However, its role in treatment decision-making remains unclear.

**Methods:**

We conducted a retrospective study in patients ≥ 65 years old with DLBCL who were treated with R-CHOP between 2015 and 2022 (post-ACA cohort), evaluating initial dose intensity (IDI), relative dose intensity (RDI), and 2-year overall survival (OS). During this period, dose intensities of cytotoxic drugs were adjusted by the multidisciplinary oncology team according to the ACA index categories (Excellent, Good, Moderate, and Poor). Then, we compared these outcomes with a historical cohort of patients receiving R-CHOP between 2001 and 2012 (pre-ACA cohort), where the dose intensities were determined by each physician’s discretion. Propensity score matching (PSM), adjusted for the International Prognostic Index and the ACA index, was used to balance baseline characteristics between the cohorts.

**Results:**

In the post-ACA cohort comprising 135 patients with a median age of 76 years (range: 65–91 years), the median IDI, median RDI, and 2-year OS according to the aforementioned ACA categories were 97%, 80%, 70%, and 70% (*p* < 0.001); 93%, 78%, 62%, and 59% (*p* < 0.001); and 96%, 89%, 66%, and 51% (*p* < 0.001), respectively. After PSM, 98 patients from the pre- and post-ACA cohorts were paired. Despite well-balanced patient demographics, granulocyte-colony stimulating factor prophylaxis was more commonly used in the post-ACA group (100% vs. 49%, *p* < 0.001). Consequently, the post-ACA group had higher median IDI and RDI (80% vs. 68%, *p* < 0.001, and 77% vs. 47%, *p* < 0.001, respectively) and better 2-year OS (81% vs. 66%, *p* = 0.014).

**Conclusions:**

The ACA index can serve as a feasible standard-of-care framework for individualized dose intensity adjustment in older patients with DLBCL. Although this strategy organizes treatment decision-making, vulnerable patients still exhibit significantly inferior outcomes, highlighting the need for further therapeutic optimization and novel strategies.

**Supplementary Information:**

The online version contains supplementary material available at 10.1186/s12885-026-16039-6.

## Background

 Diffuse large B-cell lymphoma (DLBCL) is the most common subtype of B-cell lymphoma; however, it remains a heterogeneous disease. Cancer incidence in older patients aged ≥ 65 years is increasing due to the global rise in aging societies [1]. Historically, the standard first line treatment for DLBCL is rituximab, cyclophosphamide, doxorubicin, vincristine, and prednisolone (R-CHOP). More recently, the substitution of polatuzumab vedotin for vincristine (“Pola-RCHP”) has gained traction in use after it demonstrated a progression-free survival benefit over RCHOP [2]. However, some older patients do not tolerate R-CHOP therapy, and the incidence of cardiotoxicity and febrile neutropenia is relatively high in this population [3]. Despite being key components of R-CHOP, the dose intensities of cyclophosphamide, doxorubicin, and vincristine are occasionally reduced in older patients with DLBCL [4]. Particularly for very elderly patients aged ≥ 80 years, R-miniCHOP, consisting of approximately half doses of cytotoxic agents, is widely applied [3].

Since R-CHOP is designed as curative chemotherapy, dose reductions in cytotoxic drugs can compromise response rates [5]. Providing R-CHOP to older patients with DLBCL, particularly those considered frail, is problematic due to the risk of severe or life-threatening adverse events. However, the occurrence of these adverse events varies widely from patient to patient, even at similar ages. Recently, the utility of the simplified geriatric assessment (sGA) was demonstrated in identifying fit individuals who could benefit from standard dose R-CHOP among older patients with DLBCL aged ≥ 65 years old [6]. Many other studies have demonstrated that older patients with DLBCL who are deemed “fit” by the sGA – those who have minimal comorbid conditions and perform all activities of daily living independently – can safely receive standard-dose immunochemotherapy with survival outcomes similar to those of younger patients [7]. However, for older patients deemed unfit or frail by sGA, there is no tool to determine what dose intensity of chemoimmunotherapy to give when the standard-dose regimen is anticipated to be too toxic [8].

Previously, collaborators from the Society of Lymphoma Treatment in Japan and the West-Japan Hematology and Oncology Group proposed the Age, Comorbidity, and Albumin (ACA) index, a host-dependent prognostic model of geriatric assessment (GA) for older patients with DLBCL based on data from 836 patients aged ≥ 65 years diagnosed with DLBCL and treated with R-CHOP as initial therapy at 19 centers in Japan. Age > 75 years, Charlson Comorbidity Index (CCI) ≥ 3 points in total (excluding lymphoma), and a serum albumin level of < 3.7 g/dL were useful in predicting overall survival (OS) in older patients with DLBCL independent of the widely used International Prognostic Index (IPI) [9, 10]. Additionally, the ACA index is able to stratify the chemotherapy doses actually administered in patients with different numbers of risk factors [9].

After establishing the utility of the ACA index [11], we applied it to determine the target dose intensity for the treatment of older patients with DLBCL. We conducted an extended analysis of patients with DLBCL aged ≥ 65 years who received initial R-CHOP therapy at our related institution to explore its utility, along with the clinical outcomes of a personalized treatment strategy based on the ACA index. Additionally, we compared clinical outcomes between the post- and pre-ACA eras, using a historical cohort from the previous study.

## Methods

### Patient selection and treatment

This was a retrospective observational cohort study. Older patients with DLBCL treated with R-CHOP between August 2015 and February 2022 at the Nihon University Itabashi and Saitama Cooperative Hospitals were consecutively included in the analysis (post-ACA cohort). The inclusion criteria were a pathologic diagnosis of DLBCL (not otherwise specified [NOS]), high-grade B-cell lymphoma, NOS, or high-grade B-cell lymphoma with MYC and BCL2 and/or BCL6 rearrangements, based on the 2016 revision of the World Health Organization classification of lymphoid neoplasms [12]; age ≥ 65 years at the initiation of treatment; and intent to receive six cycles of R-CHOP for curative purposes. The exclusion criteria were refusal to participate through opt-out disclosure, insufficient clinical records, a planned number of R-CHOP cycles other than six (e.g., abbreviated cycles combined with radiation), transfer to another facility during treatment, or receiving high-dose chemotherapy for central nervous system (CNS) lesions. The age cut-off of 65 years was adopted based on internationally recognized standards for older adults and to ensure consistency with the original study of the ACA index.

The R-CHOP therapy administered at our institution consisted of rituximab (375 mg/m^2^ div. day 1), cyclophosphamide (750 mg/m^2^ div. days 1 or 2), doxorubicin (50 mg/m^2^ div. days 1 or 2), vincristine (1.4 mg/m^2^ div. days 1 or 2, capped at 2 mg/body), and prednisolone (40 mg/m^2^ p.o. days 1–5 or 2–6) for six cycles. The initial dose intensities of cyclophosphamide and doxorubicin were determined using the ACA index at the time of treatment initiation. The ACA index consisted of age (> 75 years), comorbidity (CCI score ≥ 3), and albumin (< 3.7 g/dL); using this scale, patients were categorized as either excellent (0 points), good (1 point), moderate (2 points), or poor (3 points) [9]. Since August 2015, patients considered eligible for R-CHOP had their IDI determined through discussion based on the reference target dose intensity for each ACA index category after being reviewed in a multidisciplinary lymphoma board: 100% for excellent, 80–90% for good, 60–70% for moderate, and 50% for poor. These recommendations served as local guidelines rather than a strict mandate; the final decision on the target dose intensity was made on a case-by-case basis by oncology physicians and pharmacists, considering the IPI risk and organ function. Prophylaxis for febrile neutropenia using granulocyte-colony stimulating factor (G-CSF) was performed according to the institutional protocol, based on recommendations from the American Society of Clinical Oncology (ASCO) [13].

### Clinical measurements

The major clinical outcome was estimated 2-year overall survival (OS). Secondary outcomes included initial dose intensity (IDI) and relative dose intensity (RDI) of cyclophosphamide and doxorubicin, complete response (CR) rates, incidence of unplanned R-CHOP discontinuation, febrile neutropenia (FN), and treatment-related mortality (TRM). OS was calculated from the date the initial R-CHOP regimen was started to the date of death or last follow-up. The IDI was determined as the percentage of the average first doses of cyclophosphamide and doxorubicin relative to the standard doses specified in the protocol (750 mg/m² and 50 mg/m², respectively). The RDI was defined as the percentage of the actual delivered average doses of cyclophosphamide and doxorubicin relative to the protocol-specified dose per unit of time; for example, six cycles of regular-dose drugs administered every 21 days was 100%. The response evaluation was conducted according to the Lugano Classification response criteria [14]. TRM was defined as death from any cause other than lymphoma that could be related to R-CHOP treatment. These clinical measurements were analyzed according to the four ACA index categories.

### Comparison with a historical cohort

We also performed a similar analysis using a historical cohort from the registry data of Nihon University Itabashi Hospital with similar inclusion criteria; briefly, patients aged ≥ 65 years diagnosed with de novo DLBCL, receiving at least one cycle of R-CHOP, and no CNS lesions (Pre-ACA cohort). This registry archived detailed data from 118 older patients with DLBCL treated with R-CHOP between 2001 and 2012, who were included in a previous study [9]. Subsequently, we compared the clinical outcomes of the present and historical cohorts after adjusting for confounding factors.

### Statistical methods

Categorical variables are presented as percentages (%), and differences between groups were compared using the chi-square test. Continuous variables are summarized as median values, and the Kruskal-Wallis test and Mann-Whitney U test were used for comparisons between multiple and binary groups, respectively. OS was estimated using the Kaplan–Meier method, and the log-rank test was used for comparisons. To evaluate the effects of potential confounding factors, univariate and multivariate analyses were performed using Cox proportional hazards models. Propensity score matching was used to compare the present and historical cohorts. The propensity score was calculated using a logistic regression model for ACA and IPI categories (Low/Low-intermediate and High/High-intermediate risks), which have a significant impact on OS in older patients with DLBCL [9]. Patients were then matched 1:1 using nearest-neighbor matching with a caliper of 0.2 standard deviations of the logit of the propensity score, and unmatched patients were excluded. Statistical significance was set at *p* < 0.05. Statistical analyses were performed using JMP version 18 (SAS Institute Inc., Cary, NC, USA).

## Results

### Patient characteristics

Overall, 158 patients were treated with R-CHOP during the study period; 23 were excluded because of poor records (*n* = 8), planned R-CHOP cycle number other than six (*n* = 7), transfer to other facilities during R-CHOP (*n* = 5), and administration of high-dose methotrexate for prophylaxis for CNS relapse (*n* = 3). In total, 135 patients (78 males and 57 females) with a median age of 76 years (range: 65–91 years) and a median follow-up of 3.6 years (range: 0.1–9.0 years) were included in the analysis as the post-ACA cohort. Of these, 76 (57%) were classified as having high or high–intermediate risk according to the IPI. Most patients had DLBCL, NOS (*n* = 123); however, patients with high-grade B-cell lymphoma (*n* = 6), intravascular lymphoma (*n* = 3), and transformed lymphoma (*n* = 3) were also included. No patients had evidence of CNS disease on initial evaluation. All patients were administered primary prophylaxis for FN with G-CSF (Table 1).

**Table 1:** Demographics of the post-ACA cohort

Items	Patients (*n* = 135)
Male/Female, n	78/57
> 75 years of age, n (%)	72 (53)
Albumin < 3.7 mg/dL, n (%)	67 (50)
CCI ≥ 3 points, n (%)	48 (36)
ECOG PS 2–4, n (%)	49 (36)
Elevated LDH, n (%)	84 (62)
Stage III or IV, n (%)	80 (59)
Extranodal sites (≥ 2), n (%)	40 (30)
IPI, n (%)	
High	44 (33)
High-intermediate	32 (24)
Low-intermediate	36 (27)
Low	23 (17)
ACA index, n (%)	
Excellent	27 (20)
Good	47 (35)
Moderate	43 (32)
Poor	18 (13)

*ACA* Age, comorbidity, and albumin, *CCI* Charlson Comorbidity Index, *ECOG PS* Eastern Cooperative Oncology Group Performance Status, *LDH* Lactate dehydrogenase, *IPI* International Prognostic Index

### Clinical outcomes according to ACA index status

**Fig. 1:**
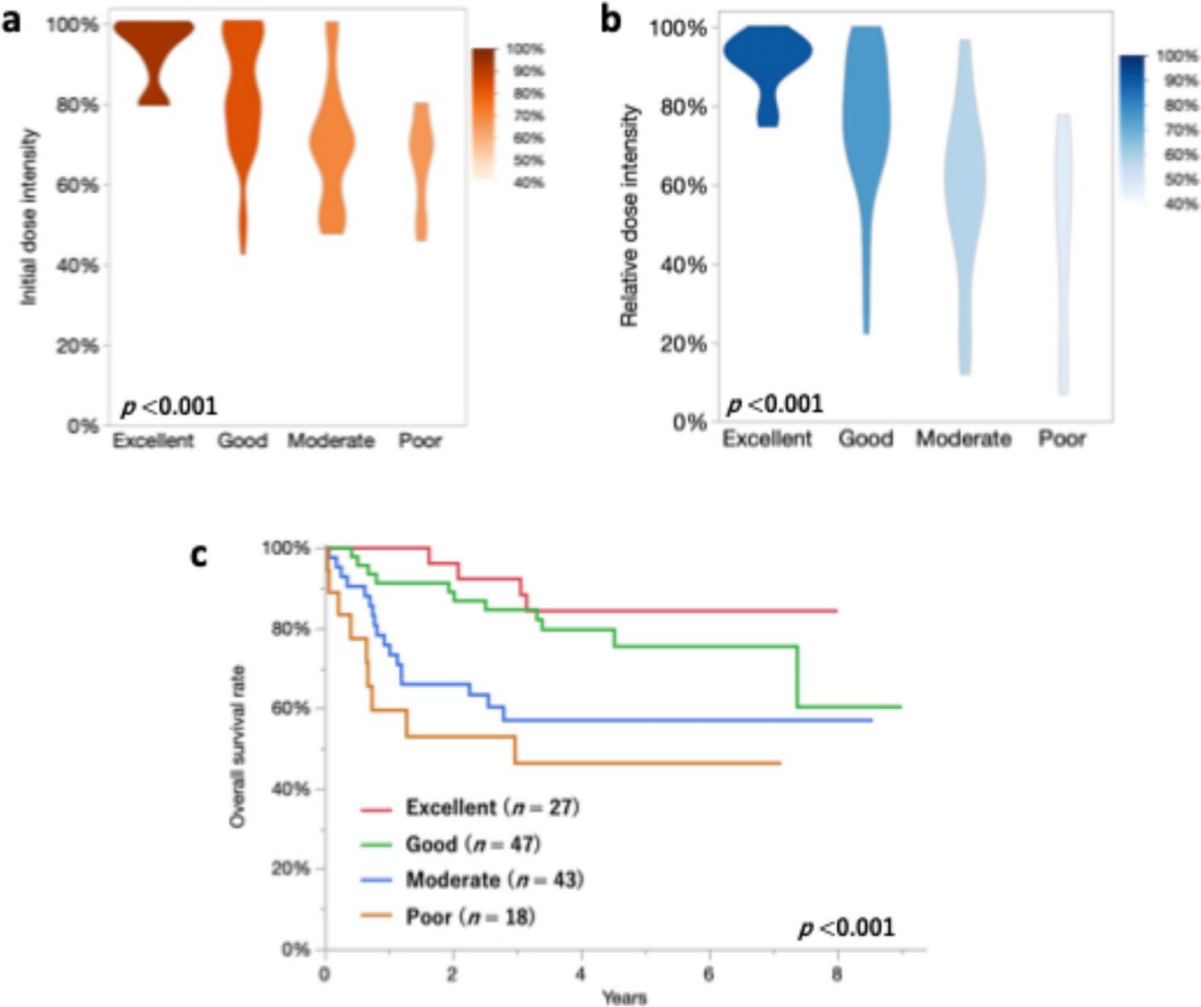
Treatment intensities and clinical outcome in the post-ACA cohort. **a** Violin plots of the initial dose intensity (IDI) and (**b**) relative dose intensity (RDI); and (**c**) Kaplan–Meier curves of overall survival, according to the ACA index category. Color gradation represents the average dose intensity

For the ACA index, 27 (20%), 47 (35%), 43 (32%), and 18 (13%) patients were classified as excellent, good, moderate, and poor, respectively. Compliance with the reference target IDI within a ± 10% range was achieved in 93 patients (69%). Among the remaining patients, the IDI was higher in 21 (16%) due to advanced disease and lower in 21 (16%) due to safety concerns. Consequently, the median group IDIs were 97%, 80%, 70%, and 70% (*p* < 0.001; Fig. 1a) and median RDIs were 93%, 78%, 62%, and 59% (*p* < 0.001; Fig. 1b). Unplanned discontinuation incidences were 4%, 12%, 26%, and 44% (*p* = 0.003); rates of FN were 7%, 11%, 14%, and 28% (*p* = 0.274), and incidences of TRM were 0%, 0%, 0%, and 17% (*p* = 0.006), respectively. Consequently, the CR rates at the end of treatment were 96%, 87%, 72%, and 50% (*p* < 0.001), and 2-year OS rates were 96%, 89%, 66%, and 51% (*p* < 0.001; Fig. 1c), respectively (Table 2). Among the 41 deaths recorded during the observation period, 29 (21%) were due to disease progression, 3 (2%) were TRM, and 9 (7%) were unrelated to either the lymphoma or the treatment. To determine the independent prognostic value of the ACA index category (Excellent/Good vs. Moderate/Poor), univariate and multivariable Cox proportional hazards analyses were performed. After adjusting for the IPI risk group (Low/Low-intermediate vs. High/High-intermediate), the ACA category had a significant impact on OS (*p* < 0.001), indicating its robust prognostic utility independent of traditional clinical risk factors in this cohort (Table 3).

**Table 2:** Clinical outcomes according to the ACA index categories in the post-ACA cohort

Items	Excellent (*n* = 27)	Good (*n* = 47)	Moderate (*n* = 43)	Poor (*n* = 18)	*p* value
Dose Intensity					
Reference target RDI (%)	100	80–90	60–70	50	-
Median IDI (%)	97	80	70	70	< 0.001
Median RDI (%)	93	78	62	59	< 0.001
Clinical Responses					
CR rate (%)	96	87	72	50	< 0.001
2-year OS (%)	96	89	66	51	< 0.001
Adverse Events					
Unplanned discontinuation (%)	4	12	26	44	0.003
Febrile neutropenia (%)	7	11	14	28	0.274
TRM (%)	0	0	0	17	0.006

*ACA* Age, comorbidity, and albumin, *RDI* Relative dose intensity, *IDI* Initial dose intensity, *CR* Complete response, *OS* Overall survival, *FN* Febrile neutropenia, *TRM* Treatment-related mortality

**Table 3:** Cox proportional hazards analysis for overall survival in the post-ACA cohort

Variables	Univariate HR (95% CI)	*p* value	Multivariate HR (95% CI)	*p* value
ACA (Poor/Moderate vs. Excellent/Good)	3.10 (1.63–6.00)	< 0.001	2.89 (1.52–5.51)	0.001
IPI (H/HI vs. L/LI) ^a^	2.96 (0.91–9.61)	0.072	2.47 (0.76–8.04)	0.134

*HR* Hazard ratio, *CI* Confidence interval, *ACA* Age, comorbidity, and albumin, *IPI* International Prognostic Index, *H/HI* High or high-intermediate risk, *L/LI* Low or low-intermediate risk

^a^As the study population consisted entirely of patients aged ≥ 65 years, the “Low” risk patients technically had a minimum IPI score of 1 due to the age factor

### Clinical demographics and findings of the historical cohort

The pre-ACA cohort consisted of 118 patients with a median age of 74 years (range: 65–90 years) and a median follow-up of 2.7 years (range: 0.1–12.0 years), including 69 men and 49 women. Of these, 42 (36%) were classified as high or high-intermediate risk according to the IPI. During this period, any type of geriatric assessment was not performed, and the target dose intensity of R-CHOP was left to the discretion of the treating physicians. As local guidelines for the use of G-CSF had not yet been established, primary or secondary prophylaxis with G-CSF was not administered in 61 patients (52%).

**Fig. 2:**
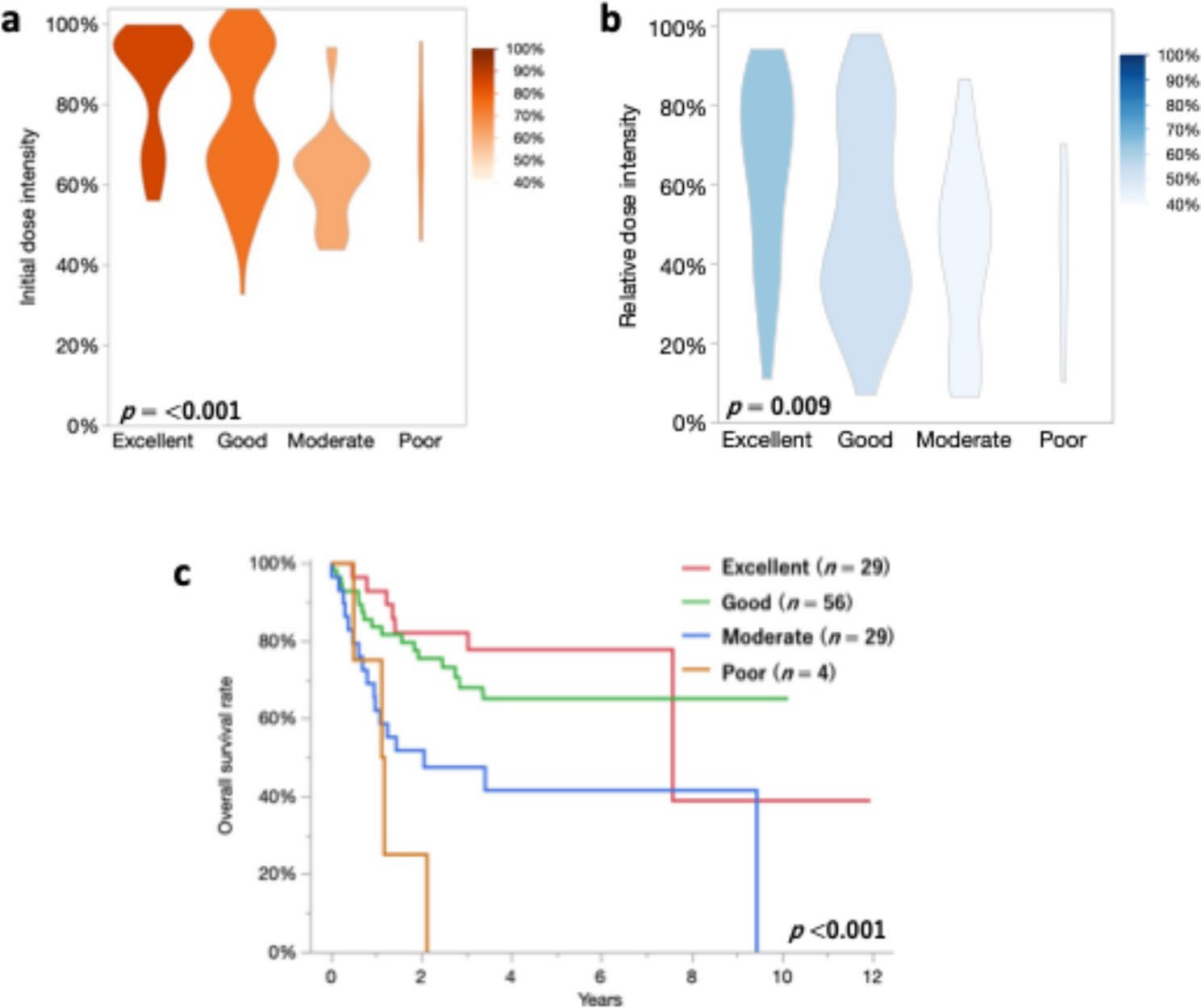
Treatment intensities and clinical outcome in the pre-ACA cohort: (**a**) Violin plots of the initial dose intensity (IDI) and (**b**) relative dose intensity (RDI); and (**c**) Kaplan–Meier curves of overall survival, according to the ACA index category. Color gradation represents the average dose intensity

The number of patients in each ACA index category was 29 (25%), 56 (47%), 29 (25%), and 4 (3%) for excellent, good, moderate, and poor, respectively. The median IDIs of each group were 93%, 70%, 63%, and 69%, respectively (*p* < 0.001; Fig. 2a), and the median RDIs were 68%, 46%, 45%, and 45% (*p* = 0.009; Fig. 2b). The incidences of unplanned discontinuation were 17%, 19%, 38%, and 25% (*p* = 0.244); those of FN were 17%, 16%, 34%, and 25% (*p* = 0.264), and those of TRM were 3%, 5%, 7%, and 0% (*p* = 0.853), respectively. Consequently, the CR rates were 79%, 80%, 51%, and 25% (*p* = 0.007), respectively, and 2-year OS rates were 82%, 75%, 52%, and 25% (*p* < 0.001; Fig. 2c). To address the potential confounding effect of G-CSF prophylaxis, we performed a sensitivity analysis on the use of G-CSF. Consequently, there was no significant difference in OS between patients who received G-CSF and those who did not (hazard ratio 0.88, 95% confidence interval [0.49–1.58], *p* = 0.669). Similarly, no significant difference was observed in median RDI between the two groups (57% vs. 45%, *p* = 0.359).

### Comparison of the present and historical cohorts

**Fig. 3:**
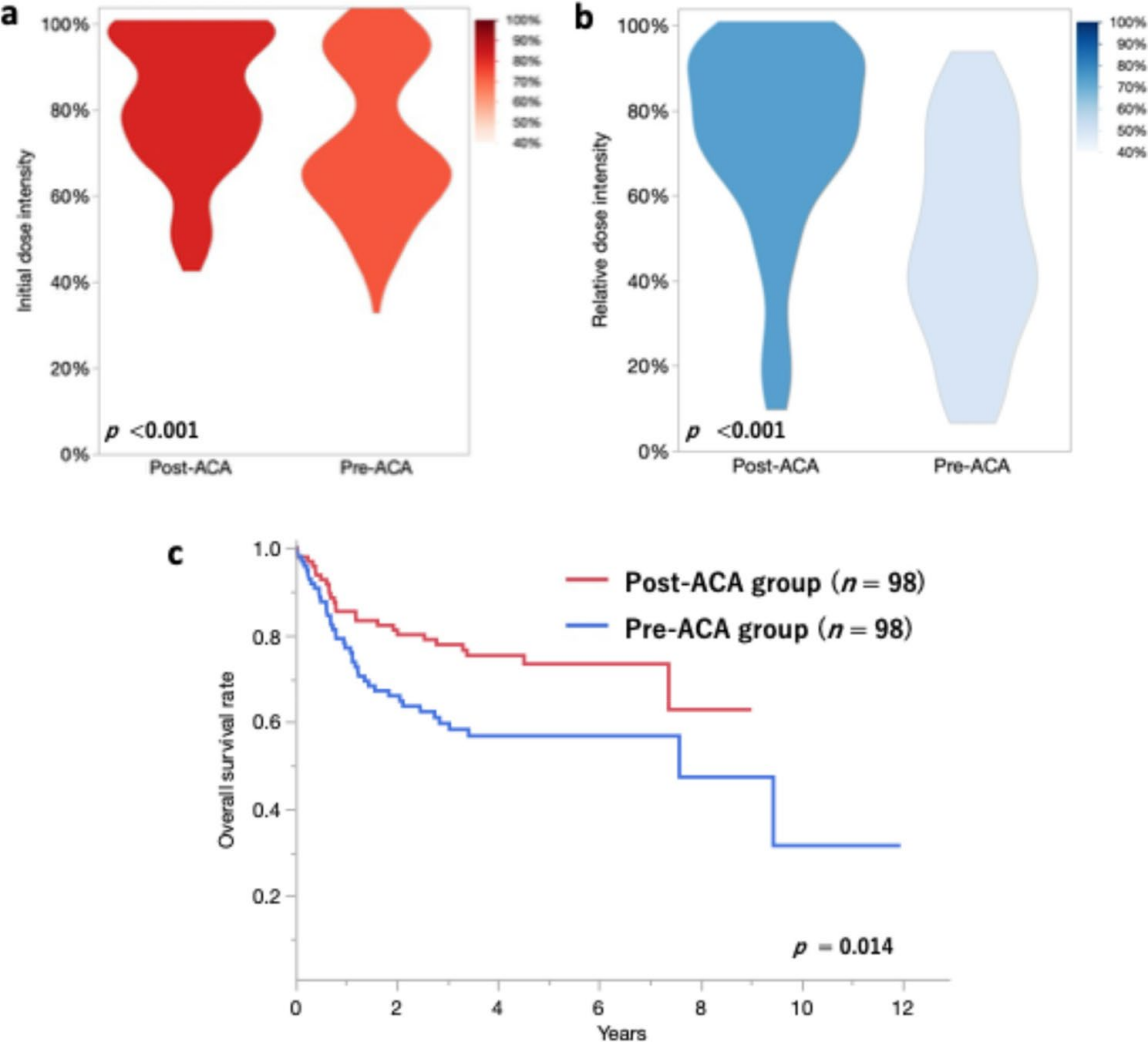
Comparison of intensities of the treatment and clinical outcomes between the post- and pre-ACA groups: (**a**) Violin plots of the initial dose intensity (IDI) and (**b**) relative dose intensity (RDI); and (**c**) Kaplan–Meier curves of overall survival. Color gradation represents the average dose intensity

After propensity score matching, 98 patients were matched from each of the post- and pre-ACA cohorts, respectively. The clinical demographics of both groups were well-balanced, excluding the proportion of patients given G-CSF prophylaxis (Table 4). Among the post- and pre-ACA groups, the median IDIs were 80% and 68%, respectively (*p* < 0.001; Fig. 3a), with median RDIs of 77% and 47%, respectively (*p* < 0.001; Fig. 2b). The incidences of unplanned discontinuation were 15% and 26% (*p* = 0.075); those of FN were 12% and 22% (*p* = 0.058), and those of TRM were 1% and 6% (*p* = 0.043), respectively. Consequently, the CR rates were 81% and 67% (*p* = 0.034), respectively, with 2-year OS rates of 81% and 66% (*p* = 0.014; Fig. 3c).

**Table 4:** Demographics of matched patients

Items	Post-ACA group (*n* = 98)	Pre-ACA group (*n* = 98)	*p* value
Median age, years (range)	73 (65–90)	74 (65–90)	0.436
G-CSF prophylaxis, n (%)	98 (100)	48 (49%)	< 0.001
High or high-intermediate IPI risk, n (%)	43 (44)	41 (42)	0.773
ACA index, n (%)			0.998
Excellent	22 (22)	23 (24)	
Good	44 (45)	43 (44)	
Moderate	28 (29)	28 (29)	
Poor	4 (4)	4 (4)	

*ACA* Age, comorbidity, and albumin, *R-CHOP* Rituximab, cyclophosphamide, doxorubicin, vincristine, and prednisolone, *IPI* International Prognostic Index

## Discussion

Here, we demonstrate the results of our ACA index-guided strategy for older patients with DLBCL and its utility in determining target dose intensities for key cytotoxic drugs in R-CHOP. In the present cohort, the IDI and RDI were well organized across the four ACA index categories. Compared to the historical cohort, patients in the present cohort in each ACA index category achieved higher median RDI and CR rates, whereas the rates of unplanned discontinuation, FN, and TRM were generally lower. Propensity score matching analysis adjusted for the ACA index and IPI revealed that the post-ACA group exhibited a significantly better CR rate and 2-year OS than those of the pre-ACA group, with significantly higher IDI and RDI and statistically marginally lower rates of unplanned discontinuation, FN, and TRM. It should be noted that the above benefits were not merely due to the ACA-guided dose adjustment of R-CHOP, as approximately half of pre-ACA patients did not receive primary FN prophylaxis with G-CSF, which is currently an evidence-based recommendation for the curative treatment of aggressive lymphomas in older adults [13]. This is largely due to the absence of local guidelines before the update of the ASCO’s recommendations in 2015; additionally, polyethylene glycolylated G-CSF products were unavailable in Japan until 2014 [13, 15]. The universal application of primary G-CSF prophylaxis in the present cohort may have contributed to their higher dose intensities and lower FN incidence. A large randomized controlled trial (RCT) revealed the benefit of primary prophylaxis with pegfilgrastim in lowering FN incidence among treated patients aged ≥ 65 years with various types of cancer, including non-Hodgkin lymphoma [16]. As for OS improvement in this population, the direct effect of G-CSF has scarcely been demonstrated, whereas a meta-analysis of 61 RCTs (irrespective of patient age or cancer type) suggested a small merit of 0.93 relative risk for all-cause mortality with G-CSF support (95% confidence interval: 0.90–0.96) [17]. However, our sensitivity analysis showed that G-CSF use did not significantly improve RDI or OS in the pre-ACA cohort. While universal G-CSF prophylaxis likely improved treatment tolerability, the marked survival benefit in the post-ACA group is considered to depend largely on the ACA index-guided strategy.

This study also reaffirms the prognostic value of the ACA index in predicting survival. It demonstrated statistically significant survival differences by ACA index group, even though the treatments were modified according to index category in the present study participants. These validations further confirm its robustness as a prognostic indicator that can discriminate OS in older patients with DLBCL with various backgrounds [18].

The scarcity of similar studies contributes to the uniqueness of this study. To date, several studies have evaluated R-CHOP-based immunochemotherapy with attenuated dose intensities for older patients with DLBCL, at a target RDI of approximately 50–75% [19–24]. However, in these trials and retrospective observations, R-CHOP dose intensities were primarily fixed, regardless of patient fitness. A limitation of these studies is that some patients may have received under- or over-treatment with such a one-size-fits-all strategy, as the tolerability of toxic treatments and required dose intensity for disease control in older patients with DLBCL are quite heterogeneous [25]. In contrast, growing evidence supports the clinical application of GA in this population. Olivieli et al. performed a multicenter prospective observational study of GA-guided therapy, which stratified older patients with DLBCL into three groups according to a simplified comprehensive GA, reporting that participants assigned to the “fit” group had favorable outcomes after standard full-dose R-CHOP [26]. Yagi et al. conducted a retrospective study of the GA-based frailty score in patients aged ≥ 70 years with DLBCL. They reported that fit patients likely benefited from standard-dose R-CHOP, while unfit and frail patients may have benefited from reduced dose intensities [27]. Additionally, larger prospective observational studies conducted by the Fondazione Italiana Linfomi have demonstrated the utility of sGA in objectively assessing the fitness of older patients with DLBCL who may benefit from standard dose immunochemotherapy [6]. Our findings demonstrate the feasibility of the ACA index-guided strategy as a standard-of-care framework for individualized dose adjustments in older patients with DLBCL. Unlike the sGA, which primarily identifies fit patients for standard-dose R-CHOP, the ACA index provides an objective tool to determine the initial dose intensity for the entire spectrum of patient fitness. This approach ensures a systematic treatment strategy even for physically unfit patients who lack clear dosing guidelines under existing assessments.

Another crucial finding of this study is that patients in the poor risk category still exhibited worse prognosis and a higher incidence of TRM, even with personalized dose adjustments. This suggests that conventional attenuated R-CHOP remains difficult to tolerate for vulnerable individuals. For such a population, novel treatment modalities with less toxic profiles should be prioritized. Currently, several clinical trials are evaluating these approaches in older or unfit patients. For example, incorporating a bispecific antibody into a non-anthracycline regimen is being investigated in this setting [28]. The ACA index could serve as a robust screening criterion to identify patients who are better suited to these innovative strategies.

This study has several limitations. First, the dose adjustment protocol was not strictly applied to all patients because the study was not designed as a prospective observational study. Some patients in the Excellent/Good ACA index group received lower doses of chemotherapy because of safety concerns, whereas some patients in the Moderate/Poor group received higher doses because of more aggressive disease. Consequently, initial dosing was outside the recommendations in 31% of patients in the post-ACA cohort, and these discrepancies may have affected our results. Additionally, despite propensity score matching, the study results were not from a direct comparison. Since the clinical context of the pre- and post-ACA eras differed, other confounding factors, such as the clinical introduction of olanzapine for antiemetic therapy or the implementation of an effective prevention protocol for rituximab infusion reactions, may have contributed to the favorable outcomes in the post-ACA group [29, 30]. However, such advancements in supportive care can be regarded as the clinical foundation for the ACA index strategy. By mitigating preventable toxicities, these measures allow the ACA index to maximize its ability to identify each biological treatment threshold of elderly patients. This ensures that fit patients receive curative intensities while protecting vulnerable individuals from the risks of excessive toxicity. Another limitation is that the methods for response evaluation differed between the cohorts. In the post-ACA cohort, most patients underwent PET/CT for response assessment according to the Lugano Classification. However, the pre-ACA cohort included cases from an era before PET/CT was widely available. Thus, a degree of bias is inevitable when comparing the CR rates between the two cohorts. Newer second-line treatment, such as polatuzumab vedotin combined with bedamustine and rituximab, may also have contributed to the favorable OS outcome in the post-ACA group [31]. Finally, based on experience from limited institutions, the study lacked external validation to generalize its results. The summary of our study and results is illustrated in Supplementary Fig. 4.

## Conclusions

In conclusion, the ACA index, a GA with a robust prognostic role in older patients with DLBCL treated with R-CHOP, has the potential to be a useful clinical tool for determining optimal dose intensity of immunochemotherapy in this globally growing population. Future large-scale observational research, such as multicenter prospective studies, is warranted to validate the general clinical utility of the ACA index.

## Supplementary Information

Supplementary Material 1

## Data Availability

Anonymized raw data of this study may be provided upon reasonable request from the corresponding author.

## Abbreviations

ACA: Age, Comorbidity, and Albumin
ASCO: American Society of Clinical Oncology
CCI: Charlson Comorbidity Index
CNS: Central nervous system
CR: Complete response
DLBCL: Diffuse large B-cell lymphoma
FN: Febrile neutropenia
GA: Geriatric assessment
G-CSF: Granulocyte-colony stimulating factor
IDI: Initial dose intensity
IPI: International Prognostic Index
NOS: Not otherwise specified
OS: Overall survival
R-CHOP: Rituximab, cyclophosphamide, doxorubicin, vincristine, and prednisolone
RCT: Randomized controlled trial
RDI: Relative dose intensity
TRM: Treatment-related mortality
