# Supplementary material for: Geriatric assessment-based therapy for older patients with diffuse large B-cell lymphoma: results of a two-decade clinical experience
Source: BMC Cancer. 2026 Apr 25;26:734. doi: 10.1186/s12885-026-16039-6 (PMC13251149; doi:10.1186/s12885-026-16039-6)
Supplement: Supplementary file 1 — Supplementary Material 1 [file 12885_2026_16039_MOESM1_ESM.docx]

**Supplementary Fig. 1 Graphic abstract of the study**

**Title:** Geriatric assessment-based therapy for older patients with diffuse large B-cell lymphoma: Results of a two-decade clinical experience

**Authors:** Hironao Nukariya^1^, Shinsaku Washinosu^2^, ﻿Haruna Nishimaki-Watanabe^3^, Takashi Ichinohe^1^, Shun Ito^1^, Toshihide Endo^1^, Kazuya Kurihara^1^, Yuichi Takeuchi^1^, Takashi Koike^1,4^, Takashi Hamada^1^, Shimon Otake^1^, Hiromichi Takahashi^1^, Masaru Nakagawa^5^, Noriyoshi Iriyama^4^, Daisuke Tsutsumi^2^, Akihiro Uchiike^2^, Tatsuya Hayama^2^, Hideki Nakamura^1^, and Katsuhiro Miura^1,2,6^

**Affiliations:** ^1^Division of Hematology and Rheumatology, Department of Medicine, Nihon University School of Medicine, Tokyo, Japan, ^2^Tumor Center, Nihon University Itabashi Hospital, Tokyo, Japan, ﻿^3^Division of Oncologic Pathology, Department of Pathology and Microbiology, Nihon University School of Medicine, Tokyo, Japan, ^4^Department of Hematology and Rheumatology, NHO Saitama Hospital, Saitama, Japan, ^5^Department of Hematology, Kasukabe Medical Center, Saitama, Japan, ^6^Department of Internal Medicine, Saitama Coopelative Hospital, Saitama, Japan
